# Editorial: The role of calcium channels in human health and disease, volume III

**DOI:** 10.3389/fmolb.2026.1803665

**Published:** 2026-02-20

**Authors:** Peng Zhang, Chang-Bo Zheng, Zhen Chen, Xiao-Yu Liu

**Affiliations:** 1 Department of Otolaryngology, Shenzhen Longgang Otolaryngology Hospital and Shenzhen Institute of Otolaryngology, Shenzhen, Guangdong, China; 2 School of Pharmaceutical Science and Yunnan Key Laboratory of Pharmacology for Natural Products, Kunming Medical University, Kunming, China; 3 Winship Cancer Institute, Emory University, Atlanta, GA, United States; 4 Shenzhen Middle School, School of Medicine, Southern University of Science and Technology, Shenzhen, Guangdong, China

**Keywords:** calcium channels, calcium signal, human disease, human health, therapeutic target

Calcium channels, situated on the cell membrane or organelle membranes, play a pivotal role in regulating the transmembrane flux of calcium ions. These channels are integral to cellular signal transduction, facilitating action potentials and neurotransmitter release in cells (Berridge, 2012). The precise regulation of calcium channels is crucial for maintaining cardiac rhythm, vascular tone, and cognitive functions such as learning and memory. Pathological conditions often involve calcium channel dysfunction, which is closely associated with arrhythmias, hypertension, epilepsy, neurodegenerative diseases, tumors, and pain (Liu et al., 2019). Consequently, understanding the regulation of calcium channels is vital for elucidating the mechanisms governing calcium homeostasis and various physiological processes. This special Research Topic presents fourteen studies exploring novel roles of calcium channels in pathophysiological contexts.

Three original research papers and three review papers in this Research Topic fconcentrated on the regulatory function of calcium channels in human cancers. Qiu et al. employed network pharmacology to identify 66 potential targets and key pathways, including calcium signaling, cAMP, and cGMP-PKG. They identified seven hub genes, and molecular docking studies confirmed their interaction with panaxadiol. Subsequent *in vitro* and *in vivo* experiments demonstrated that panaxadiol inhibits glioblastoma cell proliferation and induces apoptosis by elevating intracellular calcium levels. Tang et al. found that CDH18 was significantly overexpressed in endometrial carcinoma and correlated with poor prognosis, advanced age, and higher tumor grade. Survival analysis revealed that patients with elevated CDH18 expression exhibited shorter overall survival, and multivariate Cox regression analysis confirmed CDH18 as an independent prognostic factor. Bychkova et al. conducted an investigation into the differential effects of the bile acid 3-sulfo-taurolithocholic acid (TLC-S) on ATPase activity in human colorectal cancer (CRC) tissues and rodent livers. Their findings revealed that TLC-S significantly enhanced Na^+^/K^+^ ATPase activity in CRC tissue while reducing basal Mg^2+^ ATPase activity, effects that were not observed in the adjacent healthy colon mucosa. In contrast, in rat liver, TLC-S inhibited Na^+^/K^+^ ATPase and Ca^2+^ ATPase activity, increased cytosolic Ca^2+^ levels, and decreased mitochondrial respiration. Zhang et al. provided a systematic review of the critical roles of TRP channels in the initiation and progression of breast cancer. They emphasized that TRPC, TRPV, and TRPM channels significantly contribute to breast cancer proliferation, apoptosis, autophagy, metastasis, and angiogenesis. Lin et al. provided a comprehensive review of altered expression of voltage-gated calcium channels, TRP channels, and Orai/STIM on HNSCC. They also assessed emerging targeted therapies currently in clinical trials, such as TRP inhibitors (SOR-C13, Tranilast), T-type channel blockers (Mibefradil), store-operated calcium entry inhibitors (CAI), sarco/endoplasmic reticulum Ca^2+^-ATPase (SERCA) inhibitors (Mipsagargin), and calcium electroporation. Hu et al. examined the pivotal role of ANO1 as a calcium-activated chloride channel in both non-neoplastic conditions and neoplastic diseases. They explored the signaling pathways regulated by ANO1, such as MAPK/ERK and PI3K/PKB, proposing its significant potential as a diagnostic biomarker and a novel therapeutic target.

The dysfunctions of calcium channels play key roles in diseases related to the nervous system. Pang et al. discovered that K^+^-permeable mechanosensitive channels (K-MSCs), specifically TRAAK and BK. They coexisted with non-selective mechanosensitive channels (N-MSCs) such as TRPV4, TRPV2, and ENaC within individual neurons to mitigate pressure-induced electrical disturbances. Retinal ganglion cells demonstrate a higher relative expression of N-MSCs, leading to a reduced K/N channel ratio, which increases their susceptibility to pressure-related conditions such as glaucoma. The study suggests that modulating the level of K-MSCs relative to N-MSCs (R_K/N_ ratio) may represent a novel therapeutic strategy for neuroprotection. Yu et al. reviewed the pathways and mechanisms underlying cisplatin-induced ototoxicity, emphasizing the critical role of TRP channels. TRP channels, particularly TRPV1, facilitate cisplatin uptake and exacerbate damage through a “NOX3-ROS-TRPV1” positive feedback loop. They identified promising targeted drugs that protect hearing by modulating TRP activity. Li et al. reviewed the central role of calcium ions in cerebral ischemia-reperfusion injury and discussed related intervention strategies. During CIRI, disruption of calcium ion homeostasis leads to intracellular calcium overload, which triggers oxidative stress, inflammatory responses, enzyme activation, and cell apoptosis/necrosis, ultimately exacerbating neuronal damage.

TRPC1 and ryanodine receptor 2 (RyR2) were involved in cardiovascular disease. Lan et al. demonstrated that the absence of TRPC1 in endothelial cells exacerbates inflammation, diminishes thermogenesis, and modifies serum metabolites, thereby aggravating glucose intolerance and insulin resistance. In contrast, the overexpression of TRPC1 in endothelial cells enhances metabolic health by reducing fat accumulation and improving insulin sensitivity. The authors proposed that TRPC1 serves as a crucial regulator in vascular-metabolic coupling and represents a potential therapeutic target for metabolic syndrome. RyR2 functions as a pivotal channel governing calcium ion release in cardiomyocytes, and mutations in this receptor can result in conditions such as catecholaminergic polymorphic ventricular tachycardia (Connell et al., 2020). Zhang et al. explored how mutations in cardiac RyR2 lead to arrhythmias through a novel RyR2 structure-function parameter, specifically the interaction between the N-terminus domain and the Core Solenoid inter-subunit.

A review paper concentrated on the mechanosensitive channel Piezo1 and its involvement in calcium dynamics. They elaborated on Piezo1’s propeller-like structure and its role in transducing mechanical forces into calcium signals, which are crucial for regulating processes in vascular, skeletal, immune, nervous, and other physiological systems. Additionally, they discussed therapeutic strategies targeting Piezo1, including pharmacological modulators, gene therapy, and AI-driven approaches. One original research paper examined the role of Ca^2+^/calmodulin signaling, modulated by BaoTaiYin (BTY), in the treatment of recurrent miscarriage. Ji et al. identified 12 active constituents of BTY through the integration of UPLC-Q-TOF-MS/MS analysis, network pharmacology, and *in vitro* validation. The aqueous extract of BTY was shown to promote trophoblast proliferation by activating the IGF1R/PI3K/AKT pathway and stimulating the Ca^2+^/calmodulin signaling cascade. A review article concentrated on the role of calcium channels in the context of aging skeletal muscle. Dong et al. reviewed the impact of aging on L-type voltage-gated calcium channels, RyR1, Piezo1, TRPC/Orai1, inositol trisphosphate receptors and mitochondrial channels within skeletal muscle. These alterations disrupt calcium homeostasis, resulting in impaired excitation-contraction coupling, decreased mitochondrial metabolism, and reduced muscle regeneration, thereby contributing to the development of sarcopenia. The authors proposed potential interventions, such as physical exercise and natural bioactive compounds, to mitigate age-related channel dysfunction and muscle deterioration.

In summary, this Research Topic underscored the essential roles of calcium channels in human health and disease. Despite significant advancements in elucidating the structure and function of calcium channels, a comprehensive and systematic characterization of their roles in calcium homeostasis remains a long-term objective.
